# Correlation of anorectal manometry measures to severity of fecal incontinence in patients with anorectal malformations – a cross-sectional study

**DOI:** 10.1038/s41598-020-62908-w

**Published:** 2020-04-07

**Authors:** T. Bjørsum-Meyer, P Christensen, M. S. Jakobsen, G. Baatrup, N Qvist

**Affiliations:** 1 Department of Surgery, Odense University Hospital, Odense C, 5000 Denmark; 2 University of Southern Denmark, Faculty of Health Science, Department of Clinical research, Odense C, 5000 Denmark; 3 Department of Surgery, Aarhus University Hospital, Odense, 9000 Denmark; 4 Department of Pediatrics, Odense University Hospital, Odense, 5000 Denmark

**Keywords:** Anal diseases, Anal diseases, Pathogenesis, Pathogenesis

## Abstract

Anorectal malformations (ARM) are a spectrum of anomalies of the rectum and anal canal affecting 1 in 2500 to 5000 live births. Functional problems are common and related to the type of ARM and associated malformations. We aimed to evaluate the results of Three-dimensional High Resolution Anorectal Manometry (3D-HRAM) in long-term follow-up after surgical correction of ARM with special reference to fecal incontinence. Twenty-one patients with anorectal malformations and primary repair at our center consented to participate in the study. Pressures of the anal sphincter muscles and defects were addressed by 3D-HRAM. Fecal incontinence and disease-specific quality of life were evaluated by the Fecal Incontinence Quality of Life score and Wexner incontinence score respectively. The study was approved by the Committee in Health Research Ethics and the Danish Data Protection Agency. Median age was 22(12–31) years and 13(67%) participants were females. Sphincter defect was present in 48% (N = 10) of participants. Participants with sphincter defects had significant higher Wexner score and size of sphincter defects and mean anal squeeze pressure were correlated to Wexner score. Participants with or without sphincter defects did not differ on manometry parameters including resting anal and squeeze pressure or disease-specific quality of life. In a study of the long-term outcome after repair of anorectal malformations we found a higher Wexner incontinence score in the presence of an anal sphincter defect and the size of the defect and mean anal squeeze pressure were correlated to the Wexner incontinence score.

## Introduction

Anorectal malformations (ARM) are a spectrum of anomalies of the rectum and anal canal affecting 1 in 2500 to 5000 live births^1,2^. Even after proper reconstruction, functional problems are common and related to the type of ARM and associated malformations. Only few studies have addressed the findings on advanced anorectal manometry in relation to the long-term functional outcome^3^.

Conventional anorectal manometry has gained widely use and acceptance for the evaluation of the anorectal function in different types of anorectal malformations^4^. The results have been contradictory and with dubious clinical value. Some studies have found a correlation between the extent of sphincter damage and the degree of continence and others have not^5^. Three-Dimensional High Resolution anorectal manometry (3D-HRAM) is a technique providing an image of the pressure profile of the sphincter complex. In patients with fecal incontinence of other causes, 3D-HRAM has shown a high negative predictive value for the finding of an acquired anatomic sphincter defect compared to EUS^6^. To our best knowledge no previous studies has addressed the value of the 3D-HRAM anal manometry in the long-term follow-up after surgery for ARM.

The aim of the present study was to evaluate the results of 3D-HRAM in the long-term follow-up after surgical correction of ARM with special reference to the status of fecal continence.

## Results

### Functional results

The incidence and severity of soiling and constipation are presented in Table 1. Both soiling and constipation were present in approximately half of the participants. Only one patient experienced soiling as a social problem (grade 3). None of the participants reported constipation resistant to treatment with diet and laxatives (grade 3). The median Wexner score (interquartile range) for the 21 included participants was 4 (2–7).

**Table 1:** Incidence of bowel symptoms according to the Krickenbeck classification.

Bowel outcome measure	n(%)
Voluntary bowel movements	7(33)
Soiling	9(43)
Grade 1	7(33)
Grade 2	1(5)
Grade 3	1(5)
Constipation	11(52)
Grade 1	7(33)
Grade 2	4(19)
Grade 3	—
Soiling + Constipation	6(28)

### Manometry

In approximately half of the subjects (N = 10) a sphincter defect was detected by HRAM and in 30% the defect affected more than half of the sphincter circumference. Largest sphincter defect amounted 249 degrees and the smallest 23 degrees. Median circumferential sphincter defect was 90°. Subjects with lower ARM had significant higher anal resting pressure and anal squeeze pressure compared to subjects with intermediate ARM.

One participant was unable to distinguish between first sensation, urge and discomfort in rectal sensitivity test and in one patient first sensation and urge appeared at the same volume.

Participants with a sphincter defect, was similar to participants without sphincter defect regarding age, gender, resting pressure, squeeze pressure, rectoanal pressure gradient and HPZ as presented in Table 2. Disease-specific quality of life (FIQL) - score did not differ by any scale but Wexner score was significant higher in participants with sphincter defect.

**Table 2:** Comparison of subject with anal sphincter defect and subject without anal sphincter defect detected by High Resolution Anorectal Manometry.

Parameter	Overall	Sphincter defect	No sphincter defect	P-value
Age, years	22(12–31)	23(12–31)	22(17–31)	NS
Gender (% female)	67	60	72	NS
BMI, kg/m^2^	22.2(19.3–25.4)	19.7(18.0–31.1)	22.5(16.5–32.5)	NS
Resting anal pressure, mmHg	35(32–60)	35(32–90)	37(33–76)	NS
Anal squeeze pressure, mmHg	110(56–197)	92(16–251)	178(56–227)	NS
Rectoanal pressure gradient, mmHg	−5(−17–24)	−16(−73–88)	13(−17–41)	NS
HPZ, cm	2.6(2.5–3.7)	2.6(1.3–5.5)	3.4(2.6–4.6)	NS
FIQL score*				
Lifestyle	3.9(3.7–4.0)	3.9(2.2–4)	3.9(3.3–4.0)	NS
Coping/Behavior	3.6(3.2–4.0)	3.4(1.2–4)	3.7(3.1–4.0)	NS
Depression/Self perception	3.3(2.4–4.1)	3.3(1.9–4.3)	3.3(4.4–2.1)	NS
Embarrassment	3.3(3.0–4.0)	3.5(2.7–4)	3.3(4–1.6)	NS
Wexner score	4(0–15)	8(6–15)	2(0–7)	0.03

Data are presented as medians (interquartile range) if not otherwise indicated. HPZ: High Pressure Zone. NS: not statistical significant. *FIQL: Fecal Incontinence Quality of Life.

In participants with sphincter defect, more suffered from soiling compared to participants without sphincter defect (P = 0.009).

Participants with low malformations had lower anal resting pressure and squeeze pressure (p < 0.05). No differences were found regarding age, gender, anorectal pressure gradient, size of HPZ, appearance or size of functional sphincter defects between participants with low and intermediate malformations. One subject without a fistula and one patient with a cloaca malformation were not included in this calculation.

**Figure 1:**
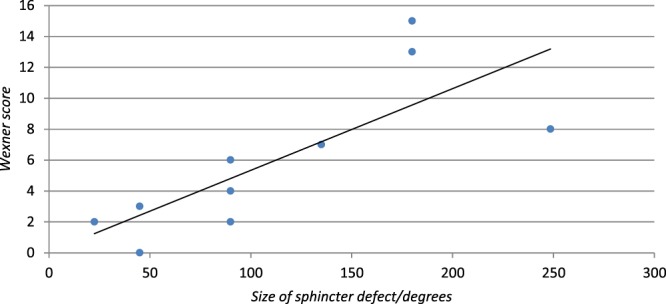
Scatterplot of Wexner vs. circumferential size of sphincter defect.

Figure 1 presents a scatterplot of Wexner score vs. circumferential size of sphincter defect. A positive and statistical significant correlation was found between Wexner score and size of sphincter defect (ρ 0.87, P 0.001). Mean Anal squeeze pressure was negatively correlated to Wexner score (ρ −0.47, P 0.04). No correlations were found regarding age (ρ 0.13 P 0.57), BMI (ρ 0.13, P 0.59), anal resting pressure (ρ −0.20, P 0.38), anorectal pressure gradient (ρ −0.28, P 0.22) or Length of HPZ (ρ −0.16 P 0.49).

## Discussion

In our study approximately half of the participants (48%) had an anal sphincter defects detected by 3D-HRAM after repair of anorectal malformations. We found size of functional sphincter defects and the mean anal squeeze pressure to be correlated to the Wexner incontinence score.

The extent of anal sphincter defect as detected by anal ultrasonography has been correlated to the severity of fecal incontinence in other populations. In a study of 250 women with a history of vaginal delivery the authors were unable to find any significant correlation between the size of an anal sphincter defect and severity of fecal incontinence^7^. The extent of the internal anal sphincter was proportionally correlated to a decrease in the mean resting anal pressure. The severity of fecal incontinence was assessed with Wexner score. The study was different from our study in only including women with a mean age of 60 years after vaginal delivery without repair of the anal sphincter. We found the extent of sphincter defect correlated to a decrease in mean anal squeeze pressure. A similar observation was found by Mion *et al*. in a study comparing 37 female patients with fecal incontinence to 36 asymptomatic volunteers and 40 constipated patients, all subjected to 3D HRAM^8^. Significantly more patients with fecal incontinence had pressure defects compared to asymptomatic controls. Patients with fecal incontinence with a median age of 62 years were significant older than both asymptomatic and constipated patients. Apart from being older patients were different from our population due to the fact that only 11 out of 37 (29%) patients had previous anorectal surgery compared to 18/21(86%) in our patients ignoring patient treated with dilatations.

Endosonography has been the gold standard for assessing the anatomical integrity of the anal sphincter after surgery for anorectal malformations^9^. The problem is that, although the structural defects may be important in the consideration of sphincter repair, the functional defects may explain the relative poor outcome in re-do procedures^10^.

In a study by Caldaro *et al*., internal anal sphincter lesions were found to be common after surgical repair of anorectal malformations. It was detected in 60% of patients with low and intermediate malformations using 3D-endoanal ultrasound (3D-EAUS)^11^. The study included 17 children after surgical repair with posterior sagittal anorectoplasty. Six out of 10 patients with low or intermediate malformations had a sphincter lesion, which they deemed to be confined to internal anal sphincter (IAS). However, an anatomic distinction between the different elements of the sphincter complex after PSARP may be difficult and the clinical importance of this is unknown especially for the minor defects. The 3D-HRAM examination gives detailed information on the function of the sphincter complex, which was correlated to functional outcome in our study. The correlation between anal defects shown by 3D-HRAM and defects by ultrasonography or MRI in patients operated for ARM has yet to be proven. We were not able to make an anatomic distinction between the different elements of the sphincter complex with 3D-HRAM but nonetheless able to correlate sphincter defects to functional outcome.

In former studies with conventional manometry a correlation between manometric parameters and clinical outcome has been found. Emblem and colleagues assessed postoperative anatomy in 40 patients with 2D - EAUS after repair of anorectal malformations and compared it to 20 healthy participants^9^. The mean age at follow-up was 16 years (range 1–22). Gender distribution was not described. Twenty-five of the 40 included patients were characterized with a high malformation. In our study only two participants were registered with high malformations. An average defect of 2.5 and 3 quadrants in the internal (IAS) and the external sphincter (EAS) was found respectively. A four level continence scoring system was applied ranging from one to four. Grade four incontinence was defined as incontinence for gas, loose and solid stool. The extent of muscle defect in the IAS was found to be correlated with the degree of incontinence.

In a study by Rezaie *et al*. 39 adult patients with fecal incontinence but with no previous history of ARM underwent EAUS and 3D-HRAM^6^. Mean age was 65 years. Thirty-one patients were females (79%) and approximately two out of three had had at least one vaginal delivery. Defects were defined as any pressure measurements below 25 mmHg equal to pressure limit for detecting anal sphincter defect in our study. The negative predictive value of HRAM in detecting sphincter defects was found to be 92%. Unfortunately there is no information on any correlation between findings from HRAM and functional outcome. In a similar study where the cut-off value for sphincter defects was a circumferential area with pressure below 10 mmHg the sensitivity and specificity of detecting EAS defects by 3D-HRAM were 65%^12^. It is generally accepted that the resting anal pressure is maintained by the IAS accounting for about 55% with the EAS and hemorrhoidal cushions adding 30% and 15%, respectively^13,14^. The anatomy and function of the pelvic flour play an important role for the size of squeeze pressure^15^. Thus, a comparison of the results of HRAM between ARM patients with other populations should be interpreted with caution. The fact that low-pressure areas on the 3D cylindrical presentation of pressure contribution as detected by HRAM may be misinterpreted as sphincter defects needs to be taken into account. Normal asymmetrical pattern of pressures in the anal canal is properly a common cause^16^. In patients with ARM hypo-development of the anal sphincter complex in different degrees is common and may alter the pressure distribution as detected by HRAM^17^.

The application of a three-dimensional probe to HRAM equipment as performed in our study has been compared to the use of a two-dimensional probe in a study by Raja *et al*. in a retrospective study of 221 patients^18^. The 3D probe enhances diagnostic gains by the identification of 29 focal anal sphincter defects not identified by 2D examinations and had a fair inter-reader agreement. It is important to identify the sphincter defects with clinical contribution in order to stratify patients to the proper treatment. Future studies in large populations need to correlate localization and extent of low-pressure areas in anal canal as detected by HRAM and perform HRAM standardized according to consensus recommendations^19^. The strength of this trial was the standardized and protocolized HRAM investigations performed by one experienced examiner. The study has some imitations. The inclusion rate was low and a possible cause of selection bias and a low statistical power. A reason for non-consenting could be that patients with anorectal malformations have been subjected to several unpleasant examinations of the anorectum during childhood and the fact that the local ethical committee only allowed invitation by letter. We assume that invitation by phone could have increased the participation rate. Although most of the patients included had a “favourable” type of anorectal anomaly, the population was heterogenic concerning age and type of repair.

The 3D-HRAM is able to provide information on the functional anatomy of the sphincter complex after reconstruction for ARM. The clinical significance of the method has yet to be proven.

## Material and methods

### Subjects

Participants were recruited from a surgical center at Odense University Hospital (OUH), Denmark with a catchment area of approximately 3.5 million people. A thorough search in local databases using the ICD-9 codes: 725.1, 725.2 (1985–1994) and ICD-10 codes: Q42 (all included) and Q438K (1995–2004) was performed. Inclusion criteria were primary surgery for anorectal malformations from 1985 to 2004 at OUH. Exclusion criterion was severe mental disability.

**Figure 2:**
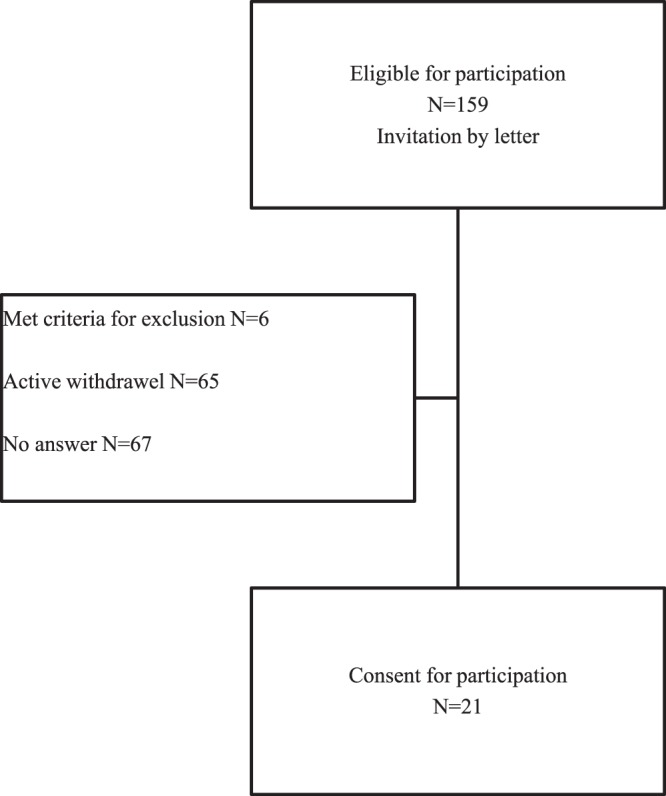
Flowchart for inclusion of participants.

Patients were invited by letter. A second invitation was send after 14 days if the subject had not answered. Twenty three subjects fulfilled inclusion criteria and consented to participate (Fig. 2). It was not possible to perform 3D-HRAM in two participants due to anal stenosis and they were therefore excluded from the analyzed population leaving 21 participants for inclusion.

In all included patients primary surgery was performed within the first year of life. There for the HRAM examination was performed at least 11 years after reconstructive anal surgery owing to the fact that the youngest participant was 12 years of age.

The following data were retrieved: age, comorbidity, type of malformation and type of primary surgery, other congenital abnormalities or malformations and in women, childbirth.

The study was performed in accordance with the 7^th^ revision of the Helsinki declaration. Prior to inclusion verbal and written informed consent was obtained. In participants below 18 years of age, the consent was retrieved from parent(s) or guardian(s). Approval was obtained from the National Committee on Health Research Ethics and the Danish Data Protection Agency.

### High resolution anorectal manometry

The manometries were performed with a Manoscan^TM^ anorectal High Resolution Manometry system (Medtronic, MN, USA). A 3D probe was mounted. The probe is composed of 256 pressure sensors circumferentially aligned over the length of 64 mm and a circumference of 10 mm. The probe was calibrated before every examination. A disposable sheath with a rectal balloon was applied to the probe and lubricated before use. Examinations were performed with the patient in the left decubitus position with hips and knees flexed 90 degrees. A rectal examination was done to ensure the rectum was empty before introducing the probe. The examinations were carried out by Author (TBM) with experience in performing anorectal manometries.

A resting period of one minute was awaited after introducing the probe before measurements. First the resting pressure was measured during a period of 20 seconds three consecutive times. The mean value was obtained. Next, Squeeze and push maneuvers were performed three times with 30 seconds intervals and mean values were used for the calculation. The recto-anal inhibitory reflex was elicited after the rectal balloon was forcefully inflated with increasing volumes of air in 10 ml aliquots. The attempt was ended at 60 ml if the reflex was not elicited. Finally, rectal sensitivity was examined. It was evaluated by inflating 10 ml of air consecutively until the patient expressed first sensation, urge to defecate and discomfort.The procedure was stopped after inflating a total volume of 400 ml.

**Figure 3:**
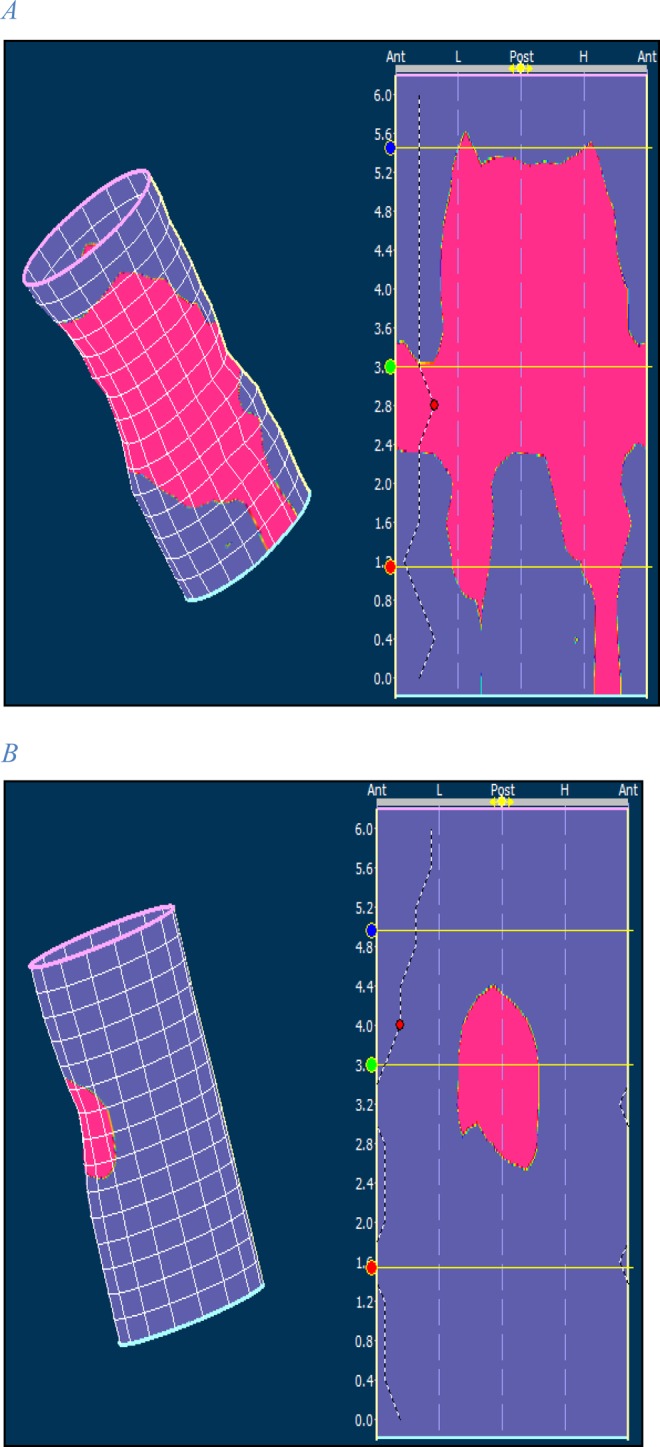
Examples of anal resting pressure profile in two participants. The 3D presentation is on the left and the 2D presentation is on the right. Pink color represents pressures equal to or above 25 mmHg. (**A**) No complete sphincter defect is present. (**B**) Large complete sphincter defect is found and only a part of the posterior sphincter complex is intact. Numbers on the left margin of the 2D presentations indicate length in centimeters. Ant: anterior. L: left. Post: posterior. H: right aspect of the sphincter complex.

The presence, size and location of a functional sphincter defect were calculated from the 3D presentation of the anal resting pressure profile (Fig. 3). We defined a functional anal sphincter defect as a pressure area below 25 mmHg and the size of the defect was calculated manually from the 3D-cylindrical pressure presentations.

### Data and statistics

Data from High Resolution Anorectal Manometry were analyzed using the guide function in the Manoview^®^ 3.0 software.

The following variables were included in the analyses: mean anal resting pressure, mean anal squeeze and maximum anal squeeze pressures, mean rectoanal pressure difference during push maneuver, high anal pressure zone, air volumes for eliciting recto-anal inhibitory reflex and air volumes for first sensation, urge to defecate and discomfort.

Data were presented as medians and interquartile ranges (IQR) if not otherwise indicated. The Fisher’s exact test was used for discrete data and for continuous data the Mann-Whitney U test was applied. A p-value below 0.05 was considered statistical significant and p-values were reported 2-sided. Spearmann’s rank order correlation was used to measure the strength and direction of association between variables and reported with a correlation coefficient rho (ρ).

### Questionnaires

Soiling and constipation were evaluated with the Krickenbeck Classification^20^ of postoperative results (Table 3). The severity of fecal incontinence was evaluated by Wexner score^21^. Disease - specific quality of life was assessed by the Fecal Incontinence Quality of Life (FIQL) - score^22^.

**Table 3:** Krickenbeck classification of postoperative results.

*Voluntary bowel movements*	Yes/no
Feeling of urge, capacity to verbalize and able to hold bowel movements.	
*Soiling*	Yes/no
Grade 1:Occassionally(once or twice per week)	
Grade 2:Every day, no social problem	
Grade 3:Constant, social problem	
*Constipation*	Yes/no
Grade 1:Manageable by changes in diet	
Grade 2:Requires laxatives	
Grade 3:Resistant to laxatives and diet	

### Subject characteristics

Subject characteristics are presented in Table 4. Of the 21 included participants five were below 18 years of age. None of the female participants have had a vaginal delivery. The anocutaneous fistula was the most common type of anomaly. In 43% (9/21) of subjects, associated anomalies appeared. Four subjects had more than one associated anomaly.

**Table 4:** Subject characteristics.

**Demographics**	
Age in years, median and (range)	22(12–31)
Female gender, N and (%)	14(67)
BMI in kg/m^2^, median and (range)	22.2(16.5–32.5)
**Type of anorectal malformation, N and (%)**	
Anocutaneous fistula	8(38)
Rectourethral fistula(bulbar)	4(19)
Rectovestibular fistula	2(10)
Rectovaginal	4(19)
Anal stenosis	1(5)
No fistula	1(5)
Cloaca	1(5)
**Associated anomalies, N and (%)**	8(38)
Vaginal septum	1
Portio duplex	1
Ventricular septal defect	1
Persistent ductus arteriosus	1
Renal agenesia	1
Hydronephrosis	1
Microencephalia	1
Clubfoot	1
**Syndromes, N and (%)**	2(10)
Trisonomi 22	1
Caudal regression syndrome	1
**Primary surgical approach, N**	
Posterior Sagittal Anorectoplasty (PSARP)	11
Perineal reconstruction	5
Dilatation	3
Abdominoperineal pull-through	1
Cutback	1

## Data Availability

Further data on demographics and manometry are available upon request from corresponding author.
